# Resveratrol-Laden Nano-Systems in the Cancer Environment: Views and Reviews

**DOI:** 10.3390/cancers15184499

**Published:** 2023-09-10

**Authors:** Muhammad Sarfraz, Mosab Arafat, Syeda Huma H. Zaidi, Lina Eltaib, Muhammad Irfan Siddique, Mehnaz Kamal, Abuzer Ali, Syed Mohammed Basheeruddin Asdaq, Abida Khan, Shams Aaghaz, Mohammed Sanad Alshammari, Mohd Imran

**Affiliations:** 1 College of Pharmacy, Al Ain University, Al Ain Campus, Al Ain P.O. Box 64141, United Arab Emirates; 2 Department of Chemistry, Faculty of Science, Northern Border University, Arar 91431, Saudi Arabia; 3 Department of Pharmaceutics, Faculty of Pharmacy, Northern Border University, Rafha 91911, Saudi Arabia; 4 Department of Pharmaceutical Chemistry, College of Pharmacy, Prince Sattam Bin Abdulaziz University, Al-Kharj 11942, Saudi Arabia; m.uddin@psau.edu.sa; 5 Department of Pharmacognosy, College of Pharmacy, Taif University, Taif 21944, Saudi Arabia; 6 Department of Pharmacy Practice, College of Pharmacy, AlMaarefa University, Riyadh 13713, Saudi Arabia; 7 Department of Pharmaceutical Chemistry, Faculty of Pharmacy, Northern Border University, Rafha 91911, Saudi Arabiaimran.pchem@gmail.com (M.I.); 8 Department of Pharmacy, School of Medical & Allied Sciences, Galgotias University, Greater Noida 203201, India; 9 Department of Computer Science, Faculty of Computing and Information Technology, Northern Border University, Rafha 91911, Saudi Arabia

**Keywords:** polyphenol, resveratrol, nanotechnology, targeted therapy, stability, toxicity, machine learning

## Abstract

**Simple Summary:**

Resveratrol, a polyphenolic stilbene derivative, is gaining popularity in cancer treatment for its antioxidant and anti-inflammatory properties. However, its pharmacokinetic and physicochemical limitations, such as a limited water solubility, photosensitivity, and oxidative stability, limit its effectiveness. Nanotechnology has emerged as an effective tactic for molecular targeted therapy, enabling passive and active targeted techniques to improve stability, therapeutic efficacy, and reduced toxicity. This article compiles studies on resveratrol-rich nano-formulations in various cancer types, focusing on improved drug stability, higher therapeutic potential, and less toxicity to cells and tissues.

**Abstract:**

The genesis of cancer is a precisely organized process in which normal cells undergo genetic alterations that cause the cells to multiply abnormally, colonize, and metastasize to other organs such as the liver, lungs, colon, and brain. Potential drugs that could modify these carcinogenic pathways are the ones that will be used in clinical trials as anti-cancer drugs. Resveratrol (RES) is a polyphenolic natural antitoxin that has been utilized for the treatment of several diseases, owing to its ability to scavenge free radicals, control the expression and activity of antioxidant enzymes, and have effects on inflammation, cancer, aging, diabetes, and cardioprotection. Although RES has a variety of pharmacological uses and shows promising applications in natural medicine, its unpredictable pharmacokinetics compromise its therapeutic efficacy and prevent its use in clinical settings. RES has been encapsulated into various nanocarriers, such as liposomes, polymeric nanoparticles, lipidic nanocarriers, and inorganic nanoparticles, to address these issues. These nanocarriers can modulate drug release, increase bioavailability, and reach therapeutically relevant plasma concentrations. Studies on resveratrol-rich nano-formulations in various cancer types are compiled in the current article. Studies relating to enhanced drug stability, increased therapeutic potential in terms of pharmacokinetics and pharmacodynamics, and reduced toxicity to cells and tissues are the main topics of this research. To keep the readers informed about the current state of resveratrol nano-formulations from an industrial perspective, some recent and significant patent literature has also been provided. Here, the prospects for nano-formulations are briefly discussed, along with machine learning and pharmacometrics methods for resolving resveratrol’s pharmacokinetic concerns.

## 1. Introduction

Polyphenols are among the most widely used active natural chemicals due to their bioavailable characteristics and potential health benefits, such as antioxidant, anti-inflammatory, anti-aging, and anti-cancer capabilities [1,2,3,4,5,6]. As oxidative stress effectors, polyphenols have been thoroughly researched concerning cancer. The topical administration of nano-polyphenols is considered to be a viable solution for circumventing various constraints in pharmacokinetics, targeted efficacy, and safety issues, although their low stability is a major bottleneck. Resveratrol (RES) has emerged as a prominent research focus among this diverse group of chemicals because of its potent anti-cancer and antioxidant properties [7,8].

**Figure 1:**
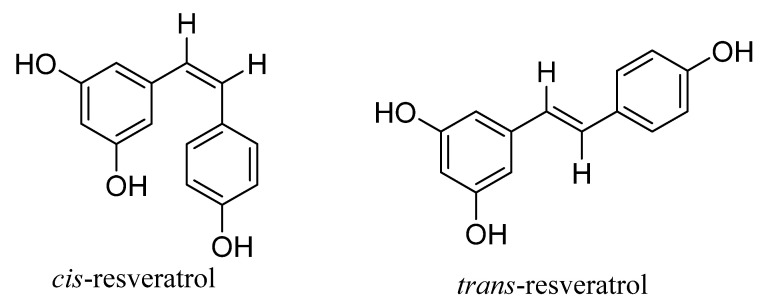
*cis–trans* isomers of resveratrol.

Chemically, RES exists in both cis and trans (Figure 1) versions, with the trans form being the most stable and therapeutically active of the two [9]. Its lower water solubility of 0.03 mg/mL and susceptibility to break down when subjected to light, oxygen, temperature, and oxidative enzymes are two characteristics of RES that make it difficult to employ. Despite being isomerized into cis-RES, the molecule loses some of its initial trans-RES activity, making it less useful in various physiological and environmental situations. With its quick metabolism, RES loses some of its significance in medication use, since it is quickly cleared out of the body, which prevents the efficacy of the molecule from being preserved. Additionally, the molecule’s phenolic groups are conjugated to sulfates by intestinal enterocytes, and because of glucuronidation, its nutritional and therapeutic effects are not properly taken. The gut microbiota in the intestines hydrogenate the aliphatic trans double bonds of RES, and additional metabolites produced upon the oral administration of RES decrease the biological availability of the molecule [10]. The limitations mentioned above halt its rapid usage on the clinical front.

**Figure 2:**
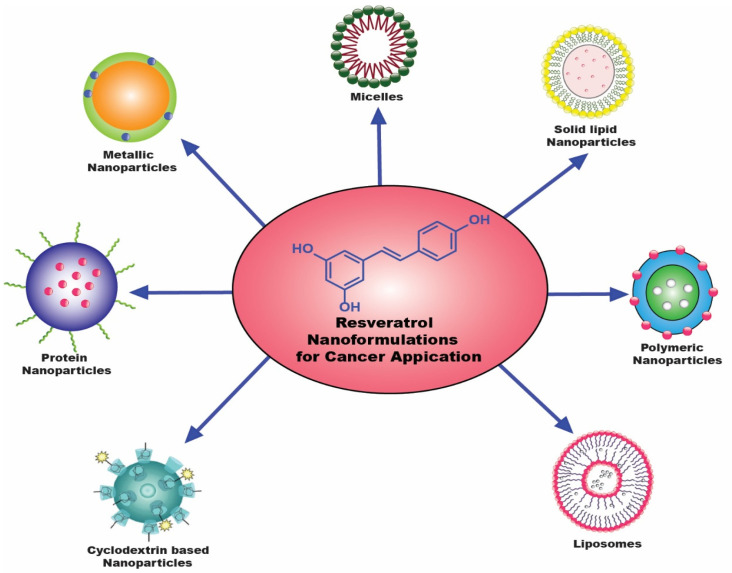
Various resveratrol-laden nanoparticles are used to treat and prevent cancer [21].

Despite being commercially marketed in several conventional dosage forms, there are not enough clinical studies on resveratrol’s effectiveness in cancer treatment and prevention [11,12]. Table 1 summarizes some of the initial-stage clinical trials on resveratrol nano-formulations. None of them were completed except NCT01476592. To overcome these limitations, nano-formulations have been developed to enhance its absorption and distribute optimal amounts of RES to the tumor target area. Some cutting-edge nano-formulations for various chemotherapy medications have revolutionized healthcare [13,14,15,16,17]. Figure 2 depicts the routinely employed nanocarriers for resveratrol delivery. Resveratrol’s bioavailability, metabolism, and transport have all been enhanced by nanomedicines [18,19]. They also have benefits such as increased tumor targeting, better solubility, and chemical stability for resveratrol [20].

The superior efficacy of nano-formulations with few side effects is achieved by an increased aggregation in tumor tissues and a reduced persistence in healthy tissues [22,23]. Moreover, through surface modification, nanoparticles have been coupled with various targeting ligands, including peptides, antibodies, and aptamers, allowing for the direct delivery of the load to the designated tumor with an increased efficacy and decreased toxicity [24,25,26,27].

Research on resveratrol-laden nano-formulations in various cancer types is compiled in the current article. It primarily focuses on research relevant to studies pertaining to a better drug stability, an increased therapeutic potential in terms of pharmacokinetics and pharmacodynamics, and a reduced toxicity to cells and tissues.

## 2. Mode of Action of Resveratrol

**Figure 3:**
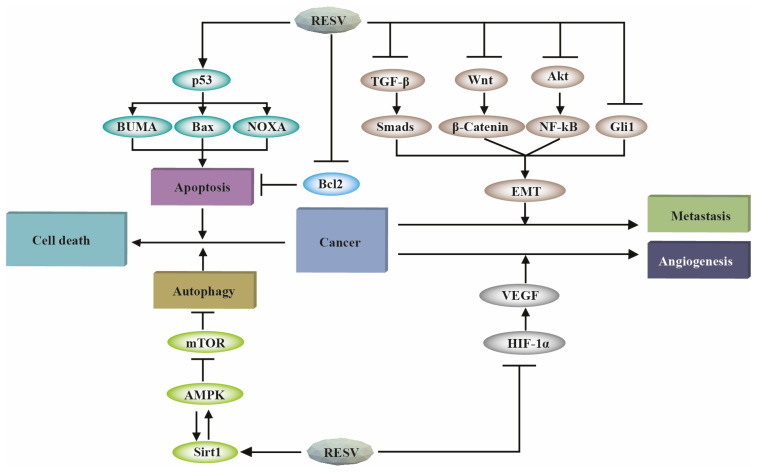
Common mechanisms through which resveratrol slows the spread of cancer. The figure has been reproduced with permission [33]. Abbreviations: RES—resveratrol, p53—tumor protein P53, PUMA—p53 upregulated modulator of apoptosis, Bax—Bcl-2-associated X protein, NOXA—phorbol-12-myristate-13-acetate-induced protein 1, Bcl2—B-cell lymphoma 2, TGF-β—transforming growth factor beta, Wnt—wingless-related integration site, Akt—also known as protein kinase B, Smads— suppressor of mothers against decapentaplegic, NF-κB—nuclear factor kappa B, Gli1—glioma-associated oncogene family zinc finger-1, EMT—epithelial–mesenchymal transition, HIF-1α—hypoxia-inducible factor 1-alpha, mTOR—mammalian target of rapamycin, AMPK—AMP-activated protein kinase, and Sirt1—sirtuin 1.

Several pathways are involved in the putative modes of action by which RES exhibits its cytotoxic activity [28,29]. Resveratrol’s capability to inhibit the processes of metastasis and angiogenesis, as well as its ability to trigger cancer cell death through apoptosis and autophagy, are the various mechanisms through which resveratrol exhibits its anti-cancer action. Table 2 represents some of the mechanisms by which resveratrol halts the progression of cancer cells. By activating the pro-apoptotic proteins of the p53 family (i.e., Bax, NOXA, and BUMA), resveratrol causes cancer cells to die. Additionally, resveratrol promotes Sirt1 and AMPK expression, resulting in cancer cells’ autophagic demise. Resveratrol prevents metastasis by inhibiting the EMT and downregulating the TGF-β1/Smads, Wnt/β-catenin, PI3K/Akt/NF-κB, and Gli1 pathways. Resveratrol prevents angiogenesis by inhibiting VEGF in an HIF-α1-dependent manner (Figure 3).

**Table 2:** Mechanism of action of resveratrol leading to diminution of cancer cell growth.

Mode of Action	Description	Ref.
Inhibition of metastasis	Decreases the expression levels of MMP-2 and MMP-9, fibronectin, and α-smooth muscle actin (α-SMA)	[30]
Inhibition of angiogenesis	Inhibition of endothelial cell adhesion and migration by reducing MMP-2 activity during neo-angiogenesis	[31]
Induction of autophagy	Increased activation and expression of sirtuin1 (SIRT1) and the inhibition of the protein kinase B/mammalian target of rapamycin (Akt/mTOR)	[32]
Induction of apoptosis	Activation of the extracellular signal-regulated kinase (ERK)1/2 via MAPK-kinase	[33]
Reprogramming of cancer cell metabolism	Regulates the enzymatic activity of pyruvate dehydrogenase (PDH) in obtaining coenzyme A	[34]

While the exact mechanisms of action of resveratrol’s anti-cancer activity are still being studied, several potential mechanisms have been proposed here:

### 2.1. Resveratrol-Mediated Induction of Autophagy

During stress, injury, aging, and pathogen infection, autophagy is a critical reaction to the cellular surroundings and positively modulates the cellular mechanisms for survival or death [35]. Autophagy is a mechanism that eliminates long-lived, compromised, and abnormal organelles and proteins from healthy tissues. However, relying on the cellular milieu can prevent or accelerate cancer cell proliferation [36]. Another method for eliminating tumor cells, particularly in cells with apoptotic defects, is the therapeutic activation of autophagic cell death through excessive autophagy [37]. Through the SIRT1/AMPK-dependent suppression of mTOR, resveratrol promotes the process of autophagy [38]. By promoting autophagy, resveratrol has been demonstrated to cause cell death in several malignancies. According to Lang et al. [39], autophagy plays a role in the partial mediation of resveratrol-induced apoptosis in human ovarian cancer (OVCAR-3) cells.

Additionally, it has been demonstrated that resveratrol causes autophagy and death in cisplatin-resistant human oral cancer (CAR) cells by promoting the expression of autophagic proteins such as Atg5, Atg12, Beclin-1, and LC3-II [40]. According to reports, autophagy is used by cancer cells as a form of defense against anti-cancer therapy [41]. In T-cell acute lymphoblastic leukemia (T-ALL) cells, resveratrol has been shown to promote both autophagy and apoptosis in a dose- and time-dependent fashion [42]. However, suppressing autophagy renders TALL cells more sensitive to resveratrol-induced apoptosis.

### 2.2. Resveratrol-Facilitated Induction of Apoptosis

Resveratrol promotes apoptosis in various cancer cells, according to lines of proof. However, its precise mechanism varies significantly depending on the type of cancer cell. According to reports, it stimulates cell growth. It has been discovered that resveratrol activates the pyruvate dehydrogenase complex (PDHc) inside cancer cells to counteract the Warburg effect. Additionally, resveratrol inhibits glycolysis, cell cycle arrest, and apoptosis within cancer cells by deactivating hexokinase II (HK II) and activating P53, and it has also been discovered to trigger apoptosis in P53-mutant tumor cells [43]. These results imply that both P53-dependent and P53-independent mechanisms can lead to apoptosis in the presence of resveratrol.

### 2.3. Resveratrol-Assisted Inhibition of Angiogenesis

Resveratrol has been demonstrated to inhibit angiogenesis by controlling VEGF (vascular endothelial growth factor). A powerful mitogen that is unique to endothelial cells, VEGF is essential for the process of tumor angiogenesis. Resveratrol has been shown to inhibit osteosarcoma cells from expressing VEGF [44]. Additionally, it reduces hypoxia-inducible factor 1 (HIF-1) expression in human ovarian cancer cells [45]. It is noteworthy that resveratrol not only inhibits the basal expression of HIF-1 and its stimulation by IGF-I under the control of Akt and MAPK, but it also promotes the proteasomal breakdown of HIF-1 [46].

### 2.4. Resveratrol-Enabled Inhibition of Metastasis

The intricate process of metastasis entails disseminating cancer cells from the initial tumor site to distant tumor-forming sites. The epithelial-to-mesenchymal transition (EMT), which occurs in the first stage of the metastatic cascade, is essential. According to research, resveratrol blocks several signaling pathways that control the EMT, reducing cancer cells’ ability to spread and move around. Resveratrol is thought to prevent the metastasis of CRC to the liver and lungs by downregulating the TGF-1/Smads pathway, which elevates the expression of E-cadherin and lowers the expression of vimentin [47]. Additionally, resveratrol inhibits CRC invasion and metastasis by downregulating the long non-coding, metastasis-associated lung adenocarcinoma transcript 1, suppressing Wnt/-catenin signaling [48].

### 2.5. Resveratrol-Aided Reprogramming of Metabolism in Cancer Cells

Most cancer cells, if not all of them, have altered metabolisms. Significant metabolic and carcinogenic biochemical pathway convergence has recently been found, raising the possibility of new cancer treatment strategies. Alterations in glucose metabolism, or what is known as the “Warburg effect”, were one of the first discovered and most prevalent metabolic changes in cancer cells [49].

By increasing the activity of the pyruvate dehydrogenase complex in colon cancer cells, resveratrol has been shown to reverse the Warburg effect. Additionally, it has been noted that resveratrol inhibits pancreatic stellate cells’ ability to synthesize glycolysis by downregulating miR-21 [50]. As a result, resveratrol’s targeting of miR-21-mediated glycolysis in tumor stroma may represent a fresh approach to preventing or treating clinical pancreatic ductal adenocarcinomas. Additionally, resveratrol has been shown to block glycolysis and target the AMPK/mTOR signaling pathway in ovarian cancer cells, which results in the induction of death [51].

## 3. Resveratrol-Entrapped Nanosystems

### 3.1. Enhanced Stability: In Vitro Studies

Given the prospect of dependence on the higher bioavailability of this compound, significant efforts have been undertaken to explore and improve resveratrol’s stability in vitro and in vivo since the initial indications of its therapeutic qualities were discovered. Resveratrol maintains its stability in an acidic environment (up to pH 6), although it swiftly degrades at pH 8 to 9 [52]. When heated or introduced to UV light, the transform can isomerize to the cis form. Still, this isomerization appears minimal in vivo, as only trace levels of cis-resveratrol can be found after administering it to rats and mice orally [53].

The nano-entrapment of resveratrol can boost its stability and act as a strong barrier against external elements that might lead to chemical deterioration. For instance, resveratrol-encased nanoparticles made with chitosan and poly(glutamic acid) were more stable than resveratrol, as determined by the trans-resveratrol transformation rate to its cis form [54]. For gastric cancer therapy in vitro, remarkable stability was also attained using the oligonucleotide anti-miR21 and resveratrol-loaded mesoporous silica nanoparticles functionalized with hyaluronic acid [55].

Innovative alternatives exist for lipophilic medications to be accommodated within the phospholipid bilayer and for hydrophilic pharmaceuticals to be administered in the aqueous core of liposomal nano-formulations [56]. Additionally, liposomes can inhibit different medications and biomolecules through degrading by photochemical means. For example, Coimbra et al. (2011) found that trans-chemical resveratrol’s stability was maintained when incorporated in liposomes and stored at 4 °C and 37 °C for 48 h without exposure to light [57]. Additionally, they discovered that, after 16 min of UV light exposure, 70% of the encapsulated trans-resveratrol was still intact, compared to only 10% in the pure state. When maintained in a refrigerator at 4 °C for up to two months, resveratrol-loaded liposomes have shown excellent physical stability, with no discernible modifications to the average particle size or polydispersity index [57,58]. The surface zeta (z) potential, a characteristic that makes estimates of electrostatic stabilization in a colloidal system, influences liposome stability in part [59]. A higher z-potential (positive or negative) denotes a good dispersion stability, as charged surfaces thwart electrostatic repulsion from causing liposomes to aggregate and further fuse. Diacetyl phosphate and lecithin were added to liposomal formulations to achieve negative z-potentials. For 60 days, the zeta potential and particle size were assessed to determine the durability of their formulations when stored at 4 °C. There was no discernible change in either of the two nanoscale (100 and 200 nm) measures [60]. Pickering emulsions are surfactant-stabilized emulsions, but their use is constrained by their lack of thermodynamic stability and binding capability. Hydrogel beads incorporate lipids to overcome the stated restrictions in such a situation. In a study, pH-responsive pectin hydrogel beads with resveratrol-loaded Pickering emulsions (RSV HB) were formulated using the ionic cross-linking approach to target the colon [61]. The mean particle size and size distribution demonstrated the stability of nanoparticles. The polydispersity index (PdI) is crucial for determining the width of the particle size distribution.

The mean particle size and PdI of RSV-PEs were 134.6 ± 1.97 nm and 0.102 ± 0.009, respectively. The entrapment efficiency of RSV-PEs was observed to be 96.51 ± 1.54%. The retention rate of resveratrol over time at 4 °C and 25 °C was assessed to explore the chemical stability of resveratrol. The retention rate of resveratrol was higher at 25 °C after 40 days. The outcomes showed that the resveratrol Pickering emulsion (RSV-PE) added to RSV-HB might have even lowered the resveratrol retention rate. The viscosity change and confinement effect may have been the causes of the RSV-HB’s increased stability. The loss of the water phase during the cross-linking process was driven by shrinkage, which increased the hydrogel’s viscosity above that of the sol and decreased the possibility of aggregation. RSV-HB had a pH-responsive, colon-targeting, and slow dissolution impact, according to an in vitro dissolution investigation. Additionally, in vitro digestion research has shown that RSV-HB regulates the rate of digestion and intended release. Based on these promising results, RSV-HB is a potential delivery technology for resveratrol applications that target the colon.

According to reports, SLNs increase the chemical stability of resveratrol better than liposomes. They are reported to increase the bioavailability of resveratrol while defending against chemical processes. Natural polyphenolic stilbenes such as resveratrol (RES) and oxyresveratrol (OXY) have many significant therapeutic functions. Nonetheless, the main drawbacks of their drug delivery use are their poor solubility and aqueous instability. The present study was undertaken to elucidate if resveratrol and oxyresveratrol can be enclosed in a nanosponge to increase their solubility and stability. The aqueous solubility of the said drugs is improved by the entrapment of RES and OXY in nanosponges (NS). The solubility of RES was higher than that of OXY, despite showing a low aqueous solubility. Due to the NSs’ entrapment in the cyclodextrin (CD) voids and interstitial spaces, both RES and OXY’s aqueous solubilities may have been boosted. Due to the UV light’s direct exposure, RES and OXY alone were noted to be degraded by 59.7% and 27.5%, respectively, within 15 min. In fact, it was clear that the RES-NS and OXY-NS demonstrated superior UV protection. Comparing the two drugs alone in solution, RES-NS had a two-fold protective effect, and OXY-NS had a three-fold protective effect. Both the stated molecules were protected, as RES and OXY were enclosed by NSs, which prohibited them from being exposed to the UV light [62]. A TEM microscopy analysis revealed that the nanosponges had a uniform size distribution, crystallinity, and porous nature. Additionally, the lattice planes that intersected the synthesized nanosponges suggested that the nanosponges had a high degree of crystallinity. The previous results strongly imply that nanosponges can be used as a delivery system for therapeutic compounds to increase their solubility and stability. Due to resveratrol’s (RSV) sensitivity to high temperatures, an alkaline pH, UV light, and the conversion from its trans to cis form, its use in the food sector is highly restricted. RSV has been incorporated into various lipid-based nano-carriers, such as nanoliposomes, nanoemulsions, and nanostructure lipid carriers, to address the stated constraints. Nanoliposomes (LPs), which have a hydrophilic core encircled by a lipid bilayer, are one of these and are regarded as potential lipid-based nanocarriers because they can increase the solubility, stability, and bioavailability of weakly water-soluble substances. In one reported study, researchers looked at how the size of nanoliposomes (LPs) affected resveratrol’s (RSV) solubility, antioxidant stability, in vitro release profile, Caco-2 cellular transport activity, cellular antioxidant activity, and in vivo oral bioavailability [63]. Thin-lipid film hydration and ultrasonication for 0, 2, and 10 min were used to create LPs with 300, 150, and 75 nm diameters. RSV’s solubility, in vitro release profile, cellular permeability, and antioxidant activity were all improved by the formulation of smaller-sized LPs in the range of 100 nm. An analogous trend was seen for its in vivo oral bioavailability as well. Due to their huge surface area for interacting with harsh surroundings, however, the size reduction in the RSV-loaded LPs could not support the antioxidant stability of RSV. This research advanced our knowledge of the ideal particle size range for LPs to enhance RSV’s in vitro and in vivo performance as a potent oral delivery vehicle.

### 3.2. Enhanced Therapeutic Potential

The concept that the endothelium of blood vessels grows more permeable under specific circumstances, such as inflammatory conditions or hypoxic situations, than in a healthy state is now well documented [18,64]. Rapidly expanding tumors engulf or recruit new blood vessels in response to hypoxia. These recently developed leaky capillaries provide an improved selective penetration of nanosystems and macromolecules bigger than 40 kDa to the tumor stroma [65]. Additionally, the tumor’s abnormal lymphatic outflow is a factor in the persistence of NPs. Smaller-sized drugs, which have a nearly instantaneous circulation and rapid discharge from the tumor, are exempt from this special property. As a result, incorporating small-molecule medications into nanosized drug carriers improves their pharmacokinetics, offers some tumor selectivity, and lessens adverse effects. This approach to tumor targeting is termed “passive targeting.” Furthermore, active targeting decreases the likelihood of systemic toxicity by using a chemotherapy-laden nanosystem oriented with accurate targeting ligand compounds that have a strong affinity for malignant cells due to the increased expression of receptors on the cancerous cells’ surfaces and a dwindling affinity for nearby healthy cells.

**Figure 4:**
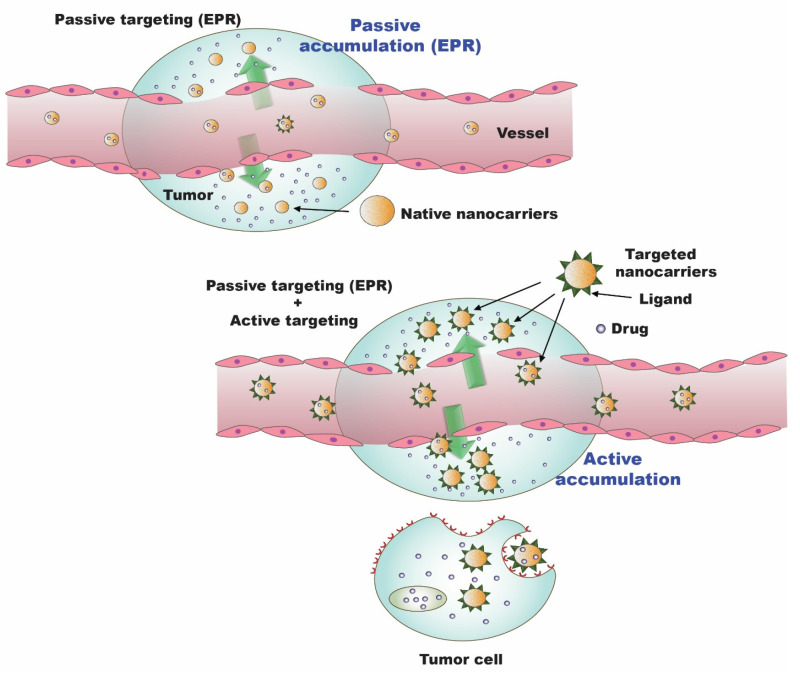
Passive and active targeting approaches in cancer therapy [66].

A range of studies have been explored to ascertain the enhanced therapeutic potential of an array of resveratrol nano-formulations employing passive and active targeting strategies, which are elaborated in this section, and the passive and active targeting approaches in cancer therapy are illustrated in Figure 4.

**Figure 5:**
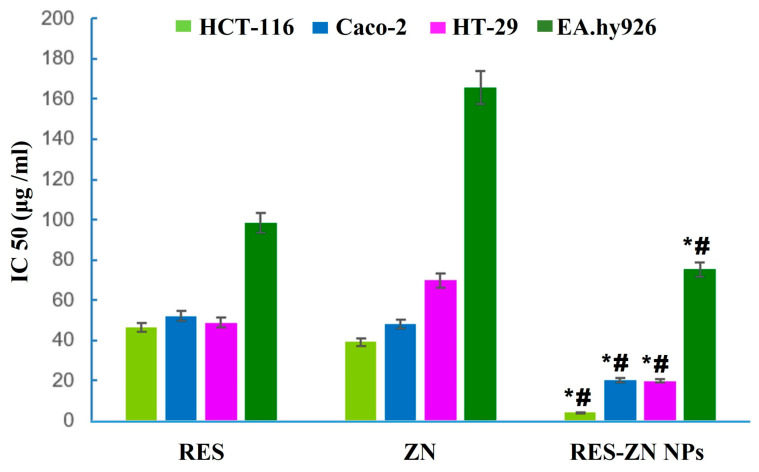
IC_50_ of RES, ZN, and ZN-RES NPs in HTC-116, Caco-2, HT-29, and EA.hy296 cell lines. * Significantly different in comparison with RES (*p* < 0.05). # Significantly different in comparison with ZN (*p* < 0.05) [67].

The prospective effects of zein nanoparticles entrapping resveratrol (RES-ZN NPs) versus RES on human colorectal cancer cells (HCT-116) in terms of antineoplastic, pro-apoptotic, and oxidative stress were investigated in a research work [67]. The chosen formula’s IC50 against HCT-116 cells was much lower than that for Caco-2 cells (Figure 5). Additionally, the RES-ZN NPs produced much more cellular uptake than the unbound RES. The higher percentage of cells in the G2 m and pre-G1 stages led to the conclusion that apoptosis was accelerated. The ZN-RES NPs caused oxidative stress biochemically, as exemplified by higher endothelial nitric oxide synthase (eNOS) isoenzyme levels and elevated ROS production. In summary, the ZN-RES NPs suppressed the cell cycle, which was backed by increased cytotoxicity, absorption, and oxidative stress indicators in the HCT-116 tumor cells compared to the free RES. These findings showed that RES’s chemopreventive profile was increased, likely due to its efficient delivery using ZN nano-dispersion against colorectal cancer.

Technetium-99m-labeled resveratrol-loaded gold nanoparticles (RES-AuNP) were fabricated [68]. The relatively high loading of resveratrol molecules was made possible by the spherical AuNPs’ larger surface area, which also increased the intermolecular association between the surface molecules and preloaded drug. The NPs were also expected to have a low reticuloendothelial intake and superior in vivo circulation time due to their spherical geometry, nanometric size range (20–30 nm), and anionic nature, along with a zeta potential of −9.1 ± 4.2 mV.

According to the studies, the 99mTc-AuNPs and 99mTc-RES-AuNPs had nontoxic behavior towards RBCs up to 100 g/mL concentrations. They showed a >70% cell survival when cultured up to a concentration of 40 g/mL for 24 h. When contrasting the biodistribution of the 99mTc-resveratrol and 99mTc-RES-AuNPs, it was speculated that the sluggish removal and decreased reticuloendothelial uptake during the initial two hours of intravenous administration were to be held responsible for the enhanced distribution of the 99mTc-RES-AuNPs in comparatively tiny, poorly perfused organs. Furthermore, 99mTc-RES-AuNPs were retained in colon tumors longer than normal colon tissue, showing that the 99mTc-RES-AuNPs showed an improved sensitivity for colon tumors. Therefore, it is postulated that the said nano-formulation can be used as a novel nanosystem for radiolabeled medications that are very effective but have a poor bioavailability, improving their internalization at the targeted site for cancer imaging purposes. Various in vivo models were used to examine the antineoplastic and anti-angiogenic effects of RES and its nano-formulation [69]. This formulation was created by incorporating RES into a polymeric nanosystem capped with chitosan. TEM micrograpy revealed the nanosystems to be in the 200–220 nm size range. The nano-formulation demonstrated a higher bioavailability and therapeutic effectiveness than the free form of the drug.

A group of researchers examined the effectiveness of resveratrol-entrapped chitosan nanoparticles (RSV-Cs NPs) in triggering apoptosis in MDA-MB 231 cells [70]. The ionic gelation technique was used to formulate dummy NPs (Cs NPs) and RSV-Cs NPs (RSV-Cs NPs), which were then further characterized. RSV, Cs NPs, and RSVCs NPs were introduced to MDA-MB 231 cells for time periods of 24, 48, and 72 h. A lactate dehydrogenase assay was used to assess the cell toxicity, and a real-time polymerase chain reaction was used to investigate the induction of apoptosis. The NPs were verified using FTIR spectra, which demonstrated cross-linking bonds. The entrapment efficiency of the RSV was 52.34 ± 0.16%, with the release occurring quickly at first and then slowly over time. The cell growth was reduced by the Cs and RSV-Cs NPs at lower concentrations and IC50 values. The strongest lethal effect was caused by the RSV-Cs NPs, which also promoted the intrinsic apoptotic pathway, as shown by upregulation of caspases 3, 8, and 9 and increases in Bcl2-associated x (BAX), BAX/Bcl2 ratio, P53 expression, and Bcl2. By focusing on the mitochondrial metabolism and activating the intrinsic apoptotic pathway, RSV-Cs NPs can significantly inhibit the proliferation of invasive breast cancer cells.

A few pieces of reported evidence have supported the growth inhibitory impact of resveratrol on breast cancer. Owing to the lower therapeutic efficacy of resveratrol, a study was conducted to formulate an ACN nanoparticle containing resveratrol to target the proliferation of breast cancer cells [71]. The outcomes showed that the encapsulation efficiency of the resveratrol nano-formulation was 87%, the particle size was 200 ± 15 nm, and the zeta potential was 31 ± 0.4 mV. The in vitro release of the produced RES + ACN demonstrated sustained release behavior. Both cell lines (MCF7 and SKBr3 cells) were more susceptible to the RES + ACN nanoparticle cytotoxicity. Enhanced expressions of Nrf2 and SOD and a greater effect on apoptosis were consistent with the decreased amount of NO and enhanced antioxidative content in both cells, particularly by the MCF7. The elevation of Nrf2 by nano-resveratrol was likely due to its interaction with ER/PR signaling factors, as seen by the decreased growth and higher expression of Nrf2 in the MCF7 cells compared to the SKBr3 cells. Still, upcoming research studies have to clarify its precise mechanism.

The Hb proportion of tumor tissue is a sign of angiogenesis progress [72]. RES drastically decreased the Hb percentages of the tumor mass, showing that it had an anti-angiogenic effect on the COLO205-luc that had been implanted. A group of researchers (Hu et al., 2019) made comparable results and discovered that RES significantly inhibited VEGF-mediated angiogenesis when used alone or in conjunction with ginkgetin [55].

RES slowed orthotopic colon tumor development and bioluminescent signals (COLO205-luc). According to the current study, cecal orthotopic transplant, Hb percentages, and tumor growth were significantly reduced in transplanted COLO205-luc by NP-RES, making it more effective than RES. This could be understood as the nano-formulation increasing the bioavailability of RSV. According to Zhao et al., Dox and RES co-entrapped in modified PLGA NPs considerably reduced the growth of Dox-resistant tumors when evaluated in MDA-MB-231 and MCF-7 tumor-bearing mice, without massively increasing systemic toxicity. Additionally, RES nanocomposites showed strong anti-cancer activity against CT26 mice colon cancer cells in vitro and a lower tissue-damaging effect [73]. Earlier in vitro investigations demonstrated that the effectiveness of RES in targeting prostate cell lines was greatly improved by polymeric NPs of RES compared to RES in its free form [74].

When administered orally, resveratrol has a low bioavailability, restricting its bioactivity. Research was undertaken to evaluate casein nanoparticles’ potential as oral resveratrol transporters [75]. Coacervation was used to synthesize the nanoparticles, which were then purified and spray-dried. The resveratrol payload was noted to be about 30 μg/mg. Studies conducted in vitro showed that pH levels did not impact the release of resveratrol from the casein nanoparticles, which followed a zero-order kinetic model. When rats were administered the nanoparticles orally, the particles persisted in the gut, showing a critical capacity to penetrate the intestinal epithelium. There was no sign of any “translocation” of the nanoparticles. The pharmacokinetic profile of the resveratrol plasma levels was analogous to that shown for the presence of the main metabolite in plasma, being high and prolonged for at least 8 h. When resveratrol was incorporated into the casein nanoparticles, its oral bioavailability was estimated to be 26.5%, which was ten times superior to when the polyphenol was given as an oral solution. Furthermore, a strong correlation was found between the in vitro and in vivo findings.

### 3.3. Toxicity Studies

Even though numerous studies have suggested that RES may exhibit many health perks and cardiovascular safeguards, in particular [76], the clinical trials conducted to date have produced conflicting results regarding the shielding effects of RES against maladies and their aftereffects [77]. The explanations for these contradictory results are numerous; however, variations in the recruited patients’ features, the doses of RES employed, and the length of the RES supplementation have been suggested as potential causes, at least in part [78]. Much research is still needed to determine the ideal RES dosage to maximize the RES health advantages while minimizing toxicity concerns [77]. It is challenging to determine the precise physiologically efficacious concentration range at which the said drug should be augmented in human subjects, because resveratrol has emerged to have a distinct dosage range in vitro as contrasted to its in vivo bioavailability. Issues have been expressed about whether achieving its in vitro efficacious concentrations in vivo would be possible. Numerous investigations have demonstrated that, in rats, RES can accumulate in particular tissues or organs at quite high concentrations, which are comparable to those employed in many in vitro investigations, even if the precise levels of the said drug in human organs and tissue are yet unknown [79]. In such a scenario, nanoencapsulation approaches come to the rescue. Various studies have been carried out worldwide, wherein the nano-entrapment of resveratrol has diminished its toxic effects. A note on the same has been presented in the following section.

Resveratrol-loaded nano-gold particles (RES-GNPs) have reportedly been developed [80]. Compared to native Res, Res-GNPs had a stronger impact on Hepg2 cells by reducing cellular proliferation and fostering apoptosis. RES-GNPs dramatically reduced the viability of the Hepg2 cells, as shown by the cell proliferation assay. It is noteworthy that the RES-GNPs’ inhibition rate was dose-dependent. In addition, the RES-GNPs and RES had IC50 values of 66.7 g/mL and 8.3 g/mL, respectively, in L02 normal cells. According to the findings, the RES-GNPs showed no overtly harmful effects on the L02 cells. Additionally, HE staining revealed that the heart, liver, kidney, and spleen had no detectable damage upon administration of the said formulation.

According to a different study, resveratrol-TPGS-SLNs had plasma half-lives that were 11.12 and 9.37 times longer than that of native resveratrol [81]. Furthermore, it was discovered that the concentration of the brain’s nanosystem was 9.23-fold greater than that of free resveratrol. According to this research, the nanocarrier could target the tumor with little buildup in the surrounding tissues, thus indicating a reduced toxicity profile.

Preclinical investigations have demonstrated the protective effects of resveratrol and its equivalents against the cardiovascular damage brought on by cancer therapy. Additionally, they have been said to have potent anti-cancer capabilities on their own and boost the anti-cancer effects of other cancer therapies. As a result, they show great promise for protecting the cardiovascular system and combating cancer [82]. They, therefore, have a great deal of potential for safeguarding the cardiovascular system and combating cancer simultaneously. Despite the highly encouraging preclinical results for resveratrol as a cardioprotective drug, many issues must be resolved before resveratrol is put through clinical trials. It is crucial to demonstrate that the same resveratrol dose may protect the heart and fight cancer, given that the compound may have hormetic dose–response qualities [83,84].

The increased use of copper oxide nanoparticles (CuONP) in various domains harms human health [85]. A study looked at the ability of resveratrol (RES) to safeguard male Wistar rats against CuONP-induced hepatotoxicity and nephrotoxicity. After seven days, it was discovered that the CuONPs, when administered intragastrically at a dose of 300 mg/kg/day to a group, caused severe hepatic and renal impairment. Treatment with resveratrol at a dose of 60 mg/kg stopped the harmful effects that the CuONPs caused. In conclusion, our research indicated that resveratrol exhibits protective activity against the harmful effects of copper oxide nanoparticles, most likely due to its antioxidant capabilities.

A group of researchers devised and created RGD-conjugated resveratrol (RES)-loaded HSA nanoparticles (HSA-RGD NPs) [86]. In a concentration-dependent approach, the targeted nano-formulations were considerably more lethal to PANC-1 cells than native RES and untargeted nano-formulations, exhibiting apoptotic morphology. While the native RES was swiftly eliminated from the blood circulation system, the systemic circulation duration of the RES was extended by the HRP-RGD NPs, rising by almost 5.43-fold. The tissue H&E staining pictographs of all the tested groups did not reveal any appreciable tissue toxicity or abnormalities, which further confirmed the suitability of the said targeted nano-formulation for biological applications.

A research study was undertaken to employ resveratrol (RES) loaded into peptide and sucrose liposomes (PSL), leading to an Res nano-formulation (PSL@RES) [87]. No behavioral anomalies or obvious indicators of dehydration linked to animal toxicity were seen during the treatment compared to the control group. Additionally, during the in vivo investigation, the mice’s body weight showed no evident reduction. However, on day 13 following treatment, the body weight was considerably lower in the RES group. The body weight measurements proved that the PSL liposome entrapment lowered the toxicity of RES. After therapy with the nano-formulation, there were no discernible inflammatory plaques or impairments to these organs on the histological images.

Furthermore, the RES-10 therapy caused severe damage to the liver tissue. A diffuse vacuolar degradation of the liver cells and peri-portal inflammatory cell infiltration were seen. These outcomes demonstrated that RES persisted in PSL liposomes, protecting the healthy organs. PSL@RES did not significantly alter the renal markers and liver enzymes. These findings supported the organ histology analyses and proved that RES is less harmful to mice when encapsulated in peptide liposomes. Table 3 shows some studies examining the toxicity of nano-formulations that trapped resveratrol.

### 3.4. New Patent Literature: Innovative Formulations and Technological Advancements

At the start of this article, it was noted that, although resveratrol is a good candidate for halting the spread of tumors, its delivery to tumor target sites is extremely challenging. Many industry–academic collaborations have been fostered to solve this issue by developing nano-formulations employing various carrier systems. For this purpose, a thorough patent search was conducted on freely available patent databases such as Espacenet, Patent Scope, and the USPTO. There were a lot of patents where resveratrol was put on multiple nano-carrier systems. This made the drug more bioavailable and less likely to break down in phase 2 of biodegradation. The nanocarrier systems discussed in the majority of patents were the nano-dendrimer–resveratrol complex, nano/microemulsions, resveratrol phospholipid composite nano-emulsion, resveratrol-loaded lecithin nanoparticles, resveratrol-loaded gold nanoparticles, resveratrol flexible liposomes, resveratrol nanoliposomes, and resveratrol nanoscale dispersoid, etc. A few relevant patents on various resveratrol nanocarrier systems are summarized in Table 4.

## 4. Challenges and Future Outlook

Resveratrol is a potent chemotherapeutic because it targets several molecular circuits and has both chemo-preventive and chemotherapeutic effects. Despite its extensive therapeutic effects, such as anti-cancer effects, resveratrol has a poor absorption due to pharmacokinetic issues Rad [114]. By overcoming numerous physicochemical, biochemical, and biotransformation-related hurdles, nanotechnology has transformed the transportation of multiple polyphenolic chemicals, including resveratrol [115]. However, several drawbacks to nano-resveratrol must be addressed, such as their toxicity, stability, and insufficient capability to load drugs [116]. Just a few secure and biocompatible polymers can be used to create nanoparticles.

The formulation of nanoparticles using a variety of natural and biocompatible polymers, the use of innovative conjugating ligands to improve cellular internalization, the use of multiple computational and mathematical modeling techniques to forecast the transit into and through tumors, and the improvement of in vivo experimentation to determine the toxic potential of nanoparticles can all be used to overcome these issues. In addition to the difficulties associated with various drug delivery strategies, nanoparticles have some drawbacks, including the need for elaborate and expensive synthetic procedures and toxic chemicals during their chemical synthesis, which could result in many environmental issues [117]. Many ethical and legal requirements must be followed to fully assess their long-term impacts on the environment, people, and animals. Consequently, it is essential to undergo post-marketing surveillance following FDA approval.

Despite the abundance of preclinical research on the chemopreventive properties of resveratrol nano-formulations, their translation to the clinic is still a long way off due to various obstacles [118]. Many clinical trials have been conducted to assess resveratrol’s pharmacokinetics, bioavailability, safety, and tolerability. Only a few of the studies mentioned have been concerned with determining if resveratrol is effective in treating particular malignancies. Trans-resveratrol, Polygonum cuspidatum (Japanese knotweed) extract, SRT501 (micronized resveratrol), resveratrol-rich seedless red grapes/grape juices (Muscadine grapes), and microencapsulated resveratrol are some of the forms of resveratrol that have been used in these studies [119]. Many malignancies, including multiple myeloma, breast cancer, follicular lymphoma, and neuroendocrine tumors, have been the subject of these trials, although most of them have examined how resveratrol affects the growth of colorectal malignancies. In reality, resveratrol has shown some promise in clinical studies on colon cancer, maybe as a result of resveratrol’s possible proximity to and extended interaction with colonic tissues. The gut epithelium also appears well-suited for absorbing nutrients and active chemicals from food and dietary constituents. Completed clinical studies can guide what should be considered when designing future clinical trials and how to direct existing trials to create the most successful treatment regimens.

The limited patient sample sizes in these clinical trials brought attention to the lack of human data on resveratrol and the pressing need for additional studies on the substance’s safety and effectiveness. The ideal resveratrol dosage has not yet been determined [120]. However, all the preliminary research provides important recommendations for subsequent clinical research so we can create the most efficient treatment plans. Moreover, in conducting clinical trials on various resveratrol-based nano-formulations, it is critical to concentrate on determining the precise mode of action of resveratrol, determining its ideal dose, and creating novel resveratrol combinations with additional medications. Focusing on a systems biology approach to examine specific global processes, developing the best formulations, finding fresh ways to combine resveratrol with other molecules, and developing a customized dosage schedule may be intelligent moves to concentrate on. Researchers must step back and conduct several additional pilot experiments, expected to produce considerably more beneficial results.

Lastly, machine learning and pharmacometrics approaches are expected to help address all these pharmacokinetic and pharmacodynamics challenges by offering predictive models, optimization algorithms, and data-driven insights [121]. These models can predict the solubility, permeability, and other physicochemical properties of resveratrol, helping in formulation screening and identifying suitable delivery systems. Machine learning algorithms can optimize formulations by considering factors such as solubility, stability, and bioavailability [122]. Nanoparticles have shown promise in enhancing the solubility and targeted delivery of resveratrol, and machine learning models can predict absorption, distribution, metabolism, and excretion properties. Targeted delivery systems can improve therapeutic efficacy while minimizing off-target effects [123].

Resveratrol’s safety and toxicity are crucial in its chemotherapeutic development. Machine learning algorithms can predict toxicity by analyzing datasets containing nano resveratrol and other nanoparticles [124]. These models assess the safety profile and identify potential risks or adverse effects. Integrating multi-omics data, such as genomics, proteomics, and metabolomics, can identify toxicity-associated molecular signatures and provide insights into biological pathways [125]. Machine learning models can predict pharmacokinetic behavior, allowing for a better understanding of nano-resveratrol’s interactions within the body. Future consideration of all these factors will probably lead to the transition of resveratrol nano-formulations from the bench to the clinical setting.

## 5. Conclusions

Establishing a platform that might react to intra- and inter-tumor heterogeneity is challenging. Neoplasms come in a wide range of morphologies, and there is a lot of heterogeneity in terms of the genetic, pathologic, and clinical characteristics of each type of tumor, which is extremely difficult for medical technology to deal with. Additionally, it has been shown that resveratrol can change several signal transduction processes. Despite having possibilities as a chemopreventive drug, resveratrol has some drawbacks, including an inadequate tumor delivery, limited chemical stability, and erratic absorption. Innovative nanostructured delivery technologies were recently developed to address these drawbacks and enhance resveratrol’s bioavailability and cellular internalization. Considering these difficulties with nano-formulations, scientists predict a rise in resveratrol nanomedicines in the years to come, due to developments in advanced and promising nano- and bioengineering techniques. Machine learning and pharmacometrics approaches to solving the issues associated with resveratrol must be extensively explored.

## Figures and Tables

**Table 1:** Clinical studies on nano-resveratrols in various cancers.

NCT Identifier	Condition	Intervention	Measures of Outcome	Status
NCT00256334	Colon cancer	Resveratrol	Test the hypothesis that resveratrol modulates Wnt signaling in vivo in colon cancer and normal colonic mucosa	Phase I (completed)
NCT02261844	Liver cancer	Resveratrol; Placebo	Improve metabolic profile of liver cells; decrease cell growth and proliferation; and decrease hepatic inflammation	Phase I/II (withdrawn due to lack of funding)
NCT00098969	Adult solid tumor	Resveratrol	Determine the concentration of resveratrol and its metabolites in healthy participants’ plasma, urine, and feces; correlate the dose with the systemic concentration of this drug and its metabolites in these participants; and determine the drug’s safety	Phase I (completed)
NCT01476592	Neuroendocrine tumor	Dietary supplement; Resveratrol	Notch1 activation in post-treatment tumor biopsy specimens when compared to pretreatment levels	Phase not mentioned (completed)
NCT04266353	Chemoprevention	Dietary supplement; Resveratrol	IGF2 assessment using ELISA assays	Phase not mentioned (suspended due to COVID-19)
NCT00433576	Adenocarcinoma of the colon; adenocarcinoma of the rectum	Resveratrol	Pharmacodynamics of resveratrol; concentrations of biomarkers	Phase I (completed)
NCT00920556	Multiple myeloma	SRT501; Bortezomib	Last Observed Response (LOR); Number of Participants with Stable Disease (SD) as Best Response (BR); Number of Participants with Stable Disease (SD) as LOR; Number of Participants with Progressive Disease (PD) PD as Best Response (BR); Time to Disease Progression; and Change From Baseline in Hematology	Phase II (withdrawn)

**Table 3:** List of studies focusing on the toxicity aspects of the resveratrol nano-formulations.

Formulation	Cancer Type	Cell Line; In Vitro Cytotoxicity	In Vivo Toxicity	Ref.
Chitosan-gellan nanofibers	Colorectal	HT29 cells; when contrasted to native resveratrol, the said nano-formulation had a nearly identical cytotoxicity profile	NA	[88]
Indocyanin- and folic-acid-conjugated nanoparticles	Tumor	U87 cell; cytotoxicity of targeted formulation vs. native drug: 81.4 ± 2.1% vs. 53.1 ± 1.1%	The blood measurements showed that the heart, liver, spleen, lungs, and kidneys were significantly unharmed, with selective tumor uptake; no remarkable weight loss in the studied mice	[89]
Folate-conjugated HAS nanoparticles	Liver	HePG2 cells; targeted NPs displayed a slower resveratrol release and enhanced cytotoxicity than the non-targeted formulation	No toxic effects on the major organs were marked	[90]
Piperine-loaded mixed micelles	Breast	MCF-7; superior cytotoxicity by the nano-formulation was noted	No significant tissue toxicity was noted in the heart, kidney, and lungs	[91]
Sericin nanoparticles	Colorectal	CRL-2522 and Caco-2 cells; cytotoxic action against Caco-2 cells	Not mentioned	[92]
Folic-acid-targeted micelles	Breast	MCF-7; dummy NPs displayed no cytotoxicity on the cells, and the nano-formulation sustained the release of entrapped resveratrol, leading to enhanced cytotoxicity	Reduced accumulation in the heart and kidneys due to tissue dispersion	[93]

**Table 4:** The table shows some of the most important patents on resveratrol nanocarrier systems for treating cancer and other diseases.

Patent/Application Number/Applicant	Summary
US11110068B2 (Concordia University, United States)	The patent discusses several compositions containing a dendrimer–resveratrol complex. For the treatment of various diseases, including malignancies, the composition may come in the form of a dispersion, aqueous solution, solid, semi-solid, nanosuspension, or other combinations [94].
US10882012B2 (Wakamono Corp, Vietnam)	This patent finding is connected to generating a resveratrol nano/microemulsion system. This invention might be used to treat cancers [95].
CN101579291B (Tsinghua University, China)	The invention describes a nano-emulsion composed of resveratrol and phospholipids and its preparation technique and applications. The particle size of the resveratrol phospholipid composite in this newly discovered formulation was under 200 nm. The formulation efficiently transports resveratrol to the cancer’s target site [96].
CN101579291A (Tsinghua University, China)	This invention revealed the method of the preparation of a resveratrol phospholipid composite nano-emulsion. This method produced particles with a size of less than 200 nm, which can then be applied to the treatment of cancer [97].
CN112773728A (Jiangxi Science and Technology Normal University, China)	In this discovery, the resveratrol is encased in lecithin nanoparticles to achieve the effects of continuously and gradually releasing resveratrol and the good effect of promoting the transdermal penetration of the resveratrol. This prolongs the resveratrol’s retention time on the skin and enhances its bioavailability [98].
WO2017137957A1 (Tripathi Vinaykumar, India)	This invention combines resveratrol with tree fat in a unique resveratrol delivery system. This procedure created nanoparticles with a particle size of less than 100 nm. The nanoparticles can maintain their stability and prevent phase 2 metabolism in the mammalian body [99].
CN108159427B (Southwest University, China)	The invention discloses a preparation method for gold nano-load resveratrol, in which resveratrol was facilitated to react with a chloroauric acid solution to afford the gold nano-loaded resveratrol for treating hepatic carcinoma [100].
CN108114286A (Southwest University, Chongqing, China)	The application of resveratrol in tumors is disclosed in the invention, along with the preparation of the gold nano-loaded resveratrol and its application in treating liver cancer [101]. This invention is the extended work of patent CN108159427B [100].
EP2431023A1 (Shanghai Jahwa United Company Limited, China)	The resveratrol flexible liposome is the subject of the current innovation. Due to the resveratrol flexible liposome’s strong penetrability in the current invention, the active ingredient’s transdermal absorption is significantly improved. This method could be used to topically administer chemotherapy drugs such as resveratrol [102].
CN110200829B (Hunan University of Humanities Science and Technology, China)	The application of a resveratrol nano ethosome through the skin to address bioavailability problems is the subject of the innovation. The discovery could be applied to the topical administration of resveratrol for treating skin cancer [103].
CN102614127B (Beijing Fuyuan Pharmaceuticals, China)	The resveratrol nanoscale dispersoid and a technique for making it are the subjects of the invention. When the resveratrol nanoscale dispersoid is dispersed in cold water, a transparent solution with nano-resveratrol particles smaller than 150 nanometers is obtained. The process of creating resveratrol nanoscale dispersoids with a high water dispersibility and consistency can be used to produce medications, health items, food, and cosmetics [104].
CN101214225A (Northwest A&F University, China)	The current invention describes an anti-malignant nano-emulsion containing resveratrol. The produced resveratrol nano-emulsion had particle sizes ranging from 1nm to 100 nm. The nano-emulsion had a homogeneous dispersion, high fluidity, and low viscidity. The produced nano-emulsion had a stronger anti-cancer effect and longer half-life. Moreover, the formulation mentioned above increases resveratrol’s stability by preventing the oxygenation of the compound [105].
CN111920771A (Henan University, China)	The resveratrol nano-liposome is made using the film dispersion method in this invention. This new technique claims to increase the drug’s solubility in water by about 172 times and increase its stability. It was also said to have a great encapsulation efficiency and small particle size. As per the claim, the preparation procedure is straightforward, the preparation duration is brief, the production expenses are reasonable, and industrial output can be scaled up. The applications of resveratrol in healthcare products, foods, and medicines are promoted [106].
CN105534724A (Shanghai Institute of Technology, Shanghai, China)	This invention relates to a nano-solid lipid carrier with particles of 50–200 nm, coated with resveratrol, and capable of penetrating a cuticle well. The invention did not mention the pharmacological application of this sort of nanocarrier. However, it is predicted that the formed resveratrol-coated nano-solid lipid carrier can be employed for treating skin cancer [107].
CN103040754B (Institute of Food Science and Technology of CAAS, China)	A resveratrol nano-liposome and its production method are described in this invention. The produced resveratrol nano-liposome exhibits a high encapsulation efficiency, good stability, tiny grain size, uniformity, and consistency. The resveratrol nano-liposome can be employed as a chemotherapy drug to treat liver cancer [108].
CN104688715B (Shanghai Traditional Chinese medicine hospital, China)	The subject of this invention is a solid lipid nanogranule made of resveratrol and a way to make it. The current invention’s resveratrol solid lipid nanogranules offer the following advantages: a small particle size, high bioavailability, rapid drug absorption, a high drug loading rate, ease of preparation, low toxicity, and so on [109].
CN105126116B (Changchun Institute of Applied Chemistry of CAS, China)	The current invention produces a nanoparticle form of resveratrol utilizing polyglycol monomethyl ether. The experimental results showed that the resveratrol nano-particle has good water solubility, and the resveratrol-loaded formulation better fragment tumor cells [110].
CN106954861A (Jiangsu University, China)	This discovery produces resveratrol nanoparticles with zeins incorporated using ultrasonic waves. The created zeins nanoparticles of the current invention have several benefits, including a good stability, good biocompatibility, gradual release, prolonged active period, and the capacity to be used in various industries, including food, health products, medicine, and cosmetics [111].
CN102614091B (Xia Qiang, China)	This innovation discloses a nanostructured lipid carrier for resveratrol. The resveratrol carrier system is stable and water-soluble. The mentioned formulation is prepared controllably and easily, and the method can be repeatable. Manufacturers of resveratrol-containing cosmetics can make use of this delivery system. This preparation can also effectively deliver resveratrol into the cancer microenvironment to treat various cancers [112].
KR20090132357A (Korea Food Research Institute, Korea)	In this disclosure, a nanoparticle and nano-emulsion and their synthesis methods are offered to improve the bioavailability of resveratrol, which is only marginally soluble in water. The nano-emulsions and nanoparticles may deliver resveratrol chemotherapeutics in various tumor milieus [113].

## Data Availability

The data presented in this study are available in this article.
